# Effects of sildenafil and calcitonin gene-related peptide on brainstem glutamate levels: a pharmacological proton magnetic resonance spectroscopy study at 3.0 T

**DOI:** 10.1186/s10194-018-0870-2

**Published:** 2018-06-18

**Authors:** Samaira Younis, Anders Hougaard, Casper Emil Christensen, Mark Bitsch Vestergaard, Esben Thade Petersen, Olaf Bjarne Paulson, Henrik Bo Wiberg Larsson, Messoud Ashina

**Affiliations:** 1 Danish Headache Center, Department of Neurology, Rigshospitalet Glostrup, University of Copenhagen, Copenhagen, Denmark; 2 Functional Imaging Unit, Department of Clinical Physiology, Nuclear Medicine and PET, Rigshospitalet Glostrup, University of Copenhagen, Copenhagen, Denmark; 3 Danish Research Centre for Magnetic Resonance, Centre for Functional and Diagnostic Imaging and research, Copenhagen University Hospital Hvidovre, Copenhagen, Denmark; 4 Neurobiology Research Unit, Department of Neurology, Rigshospitalet, University of Copenhagen, Copenhagen, Denmark

**Keywords:** MRS, Glutamate, Glx, Lactate, Migraine, Brainstem, Thalamus, CGRP, Sildenafil

## Abstract

**Background:**

Studies involving human pharmacological migraine models have predominantly focused on the vasoactive effects of headache-inducing drugs, including sildenafil and calcitonin gene-related peptide (CGRP). However, the role of possible glutamate level changes in the brainstem and thalamus is of emerging interest in the field of migraine research bringing forth the need for a novel, validated method to study the biochemical effects in these areas.

**Methods:**

We applied an optimized in vivo human pharmacological proton (^1^H) magnetic resonance spectroscopy (MRS) protocol (PRESS, repetition time 3000 ms, echo time 37.6–38.3 ms) at 3.0 T in combination with sildenafil and CGRP in a double-blind, placebo-controlled, randomized, double-dummy, three-way cross-over design. Seventeen healthy participants were scanned with the ^1^H-MRS protocol at baseline and twice (at 40 min and 140 min) after drug administration to investigate the sildenafil- and CGRP-induced glutamate changes in both brainstem and thalamus.

**Results:**

The glutamate levels increased transiently in the brainstem at 40–70 min after sildenafil administration compared to placebo (5.6%, *P* = 0.039). We found no sildenafil-induced glutamate changes in the thalamus, and no CGRP-induced glutamate changes in the brainstem or thalamus compared to placebo. Both sildenafil and CGRP induced headache in 53%–62% of participants. We found no interaction in the glutamate levels in the brainstem or thalamus between participants who developed sildenafil and/or CGRP-induced headache as compared to participants who did not.

**Conclusions:**

The transient sildenafil-induced glutamate change in the brainstem possibly reflects increased excitability of the brainstem neurons. CGRP did not induce brainstem or thalamic glutamate changes, suggesting that it rather exerts its headache-inducing effects on the peripheral trigeminal pain pathways.

## Background

Human pharmacological migraine models have been used for the past two decades with great success to study migraine attack mechanisms using vasoactive drugs such as calcitonin gene-related peptide (CGRP) and sildenafil [1–7]. The models have been pivotal in the development of new anti-migraine therapy [8]. Human pathophysiological studies applying these models have predominantly focused on the cerebrovascular effects of the headache-inducing substances. However, emerging evidence suggests that metabolic changes, especially of brain glutamate levels [9, 10], in the brainstem [11–15] and thalamus [15] are key processes for the initiation of migraine headache attacks and thereby potentially important effects of the headache-inducing drugs. At present, methods for the study of pharmacologically induced biochemical effects on the brainstem glutamate levels have not been validated.

Pharmacological proton (^1^H) magnetic resonance spectroscopy (MRS) provides the ability to non-invasively study drug-induced biochemical changes in the brain. Imaging of the deep brain structures, especially the brainstem by magnetic resonance imaging (MRI), is challenging due to the small size of the region of interest, location in areas of relatively high magnetic field inhomogeneity and potential physiological artifacts. Thus, it is essential to systematically investigate the quality and reproducibility of ^1^H-MRS measurements in these areas before application of the method in patients. Only a few ^1^H-MRS studies of the brainstem have previously been conducted. One such study, of patients with amyotrophic lateral sclerosis, did not report data on the reproducibility or variability of the glutamate measurements [16], while other ^1^H-MRS brainstem studies did not measure the glutamate concentrations at all [17–20] .

The headache-inducing drugs, CGRP and sildenafil, were selected for the study based on their different modes of action. CGRP is generally considered to exert its primary effect outside of the central nervous system (CNS), in the meningeal vasculature and the first order trigeminal neurons [21, 22], while sildenafil, as a lipophilic molecule, readily crosses the blood-brain barrier [23].

Here, we conducted a double-blind, placebo-controlled, randomized, double-dummy, three-way cross-over pharmacological ^1^H-MRS study to investigate the sildenafil- and CGRP-induced glutamate concentration changes in healthy participants. Our null-hypothesis was that the glutamate levels are not altered in the brainstem of healthy participants after administration of sildenafil and CGRP when compared to placebo. Additionally, we assessed the spectral quality and variability of the glutamate measurements over time in the brainstem based on our ^1^H-MRS protocol.

## Methods

### Participants

Healthy volunteers were recruited through announcement on a Danish website for recruitment of participants to health research (www.forsoegsperson.dk). Inclusion criteria were: age 18–50 years and weight 50–100 kg. Exclusion criteria were: history of any primary headache disorders (except episodic tension-type headache for < 2 day per month during the last year) according to the diagnostic criteria of the beta version of the third International Classification of Headache Disorders (ICHD-3 beta) [24], first-degree family members with migraine or other primary headache disorders according to ICHD-3 beta (except episodic tension-type headache for < 6 days per month), daily intake of medication (except oral contraceptives), no usage of safe contraception, cardiovascular, cerebrovascular, or psychiatric disease, and drug abuse. Participants were excluded if there were any contraindications to MRI such as metal implants, pacemaker, insulin pump, claustrophobia and/or surgical procedure during the last 6 weeks before inclusion. We also excluded participants with braces and teeth implants of metal, which are normally regarded MRI compatible, to avoid potential MR scan artifacts in the deep brain structures of interest.

### Experimental design

**Fig. 1:**
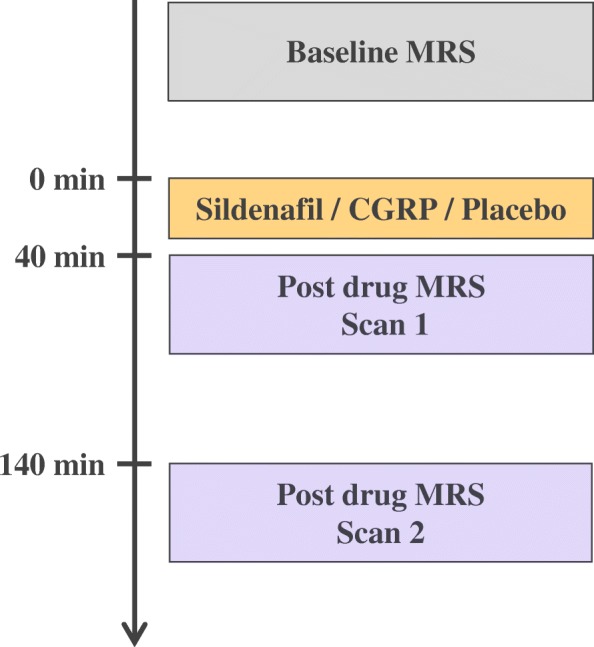
Flowchart of the study days

All participants were randomly allocated to receive sildenafil, CGRP and placebo on three separate study days. On each study day, participants underwent an MRI scan protocol consisting of three scan sessions: a baseline MRI scan, followed by two additional post drug administration MRI scan sessions. The first post drug scan was initiated at 40 min (scan 1), and the second scan was initiated at 140 min (scan 2) after administration of sildenafil, CGRP or placebo (Fig. 1). MR spectra were obtained from brainstem and thalamus during each scan.

On the sildenafil day, the participants received sildenafil as two 50 mg tablets (STADA, Bad Vilbel, Germany) in two non-transparent capsules, combined with placebo isotonic saline infusion into the cubital vein for 20 min (Pressure tubes, Argon Medical Devices, The Hague, the Netherlands), at the time of infusion start. On the CGRP day, the participants received 1.5 μg/min human-alfa-CGRP (PolyPeptide, Strasbourg, France) via infusion for 20 min combined with placebo calcium in two non-transparent capsules. On the placebo day, the participants received placebo isotonic saline infusion for 20 min combined with placebo calcium in two non-transparent capsules. The sildenafil and CGRP dosages for the study were determined based on findings of previous studies, which reported sildenafil- and CGRP-induced headache in healthy volunteers, and migraine-like attack in migraine patients [1–7]. The randomization was administrated by the Hospital Pharmacy of the Capital Region of Denmark.

All participants were headache-free for at least 72 h before each study day. The participants were not allowed coffee, tea, cocoa, soft drinks, alcohol or tobacco for 12 h before study start on each study day, and fasted for all food and beverages (except for water), for 4 h before study start. Between scan 1 and scan 2, all participants were offered a standardized small meal consisting of soft bread with cheese, banana, and water. Other criteria for the study days were no intake of any medication four half-lives before the start of the study day, except for oral contraception. After insertion of a peripheral venous catheter (18G Vasofix® Safety, B.Braun, Melsungen, Germany) into a cubital vein, the participants were instructed to rest in a hospital bed for approximately 30 min before the baseline scans.

We aimed to initiate the scan sessions at the same time of the day on all three study days for each participant, allowing for a maximum time deviation of 1 h, to account for metabolite concentration variations due to the circadian rhythm [25, 26]. In addition, the timing of scan 1 and scan 2 was fixed according to the baseline scan. The ^1^H-MRS sequences were part of a larger study (results of these will be presented elsewhere). Before and after each scan sequence it was ensured that participants remained awake, and data were excluded in case they fell asleep during the scans as this could affect the measurements [27]. Participants were instructed to remain still and avoid any head motion during the scan sessions to ensure stable measurements from the regions of interest.

### Headache characteristics

Data on headache characteristics were acquired on each study day, i.e. intensity, quality, aggravation by physical activity, location and associated symptoms (nausea, photophobia, and phonophobia). The headache intensity was rated on a numeric rating scale ranging from 0 to 10, where ‘0’ translated to no headache and ‘10’ to the worst imaginable headache. The headache data were obtained between all scans. All participants were asked to register headache hourly in a standardized questionnaire after the last scan session until 24 h, starting from the time of study drug administration.

### Vital signs

The vital variables were registered and monitored at baseline, and during the scan sessions after study drug administration. Systolic and diastolic blood pressure were measured with an interval of 10 min, and heart rate, blood oxygen saturation and nostril end-tidal CO_2_ tension (water trap and gas sample line, Medrad, Warrendale, PA) (Veris Monitor, Medrad, Warrendale, PA) were monitored continuously.

### Data acquisition and imaging protocol

All MRI scans were performed on a 3.0 T Philips Achieva MRI scanner (Philips Medical Systems, Best, The Netherlands) using a 32-channel phase array head coil.

#### Anatomical scan

High-resolution anatomical scans were obtained with a 3D T1-weighted turbo field echo sequence (field of view 240 × 240 × 170 mm^3^; voxel size 1.00 × 1.08 × 1.10 mm^3^; echo time 3.7 ms; repetition time 8.0 ms; flip angle 8°). The reconstruction software on the scanner was used to additionally obtain the axial and coronal anatomical views of the scan to ensure correct placement of the volumes-of-interest (VOIs) for brainstem and thalamus.

#### Magnetic resonance spectroscopy

We used proton (^1^H) magnetic resonance spectroscopy (MRS) to measure the combined concentration of glutamate and glutamine (reported as ‘glutamate’), lactate, *N*-Acetylaspartate (NAA) and the total concentration of creatine i.e. phosphocreatine and creatine. The water-suppressed point-resolved spectroscopy (PRESS) pulse sequence was used in brainstem (repetition time 3000 ms; echo time 38.3 ms, voxel size 10.5 × 12.5 × 22 mm^3^; 480 acquisitions; total duration 24 min) and thalamus (repetition time 3000 ms; echo time 37.6 ms; voxel size 16 mm × 12 mm × 12 mm; 192 acquisitions; total duration 9 min 36 s). High number of acquisitions was used to ensure sufficient signal-noise-ratio. Voxel size based shimming was performed using first-order pencil beam to reduce the inhomogeneity in the chosen VOIs. The protocol was thus optimized to precisely target small VOIs in deep brain structures and to avoid cerebrospinal fluid contributions and partial volume artifacts. The repetition time was 3000 ms to ensure sufficient relaxation. The unsuppressed water signal was measured from the VOIs and used as internal reference for quantification [28]. The first VOI was placed unilaterally in the right side of the brainstem, and the second VOI was placed in the left, contralateral thalamus, following the anatomical and functional trigeminal pain pathways.

### Metabolite quantification and analysis

**Fig. 2:**
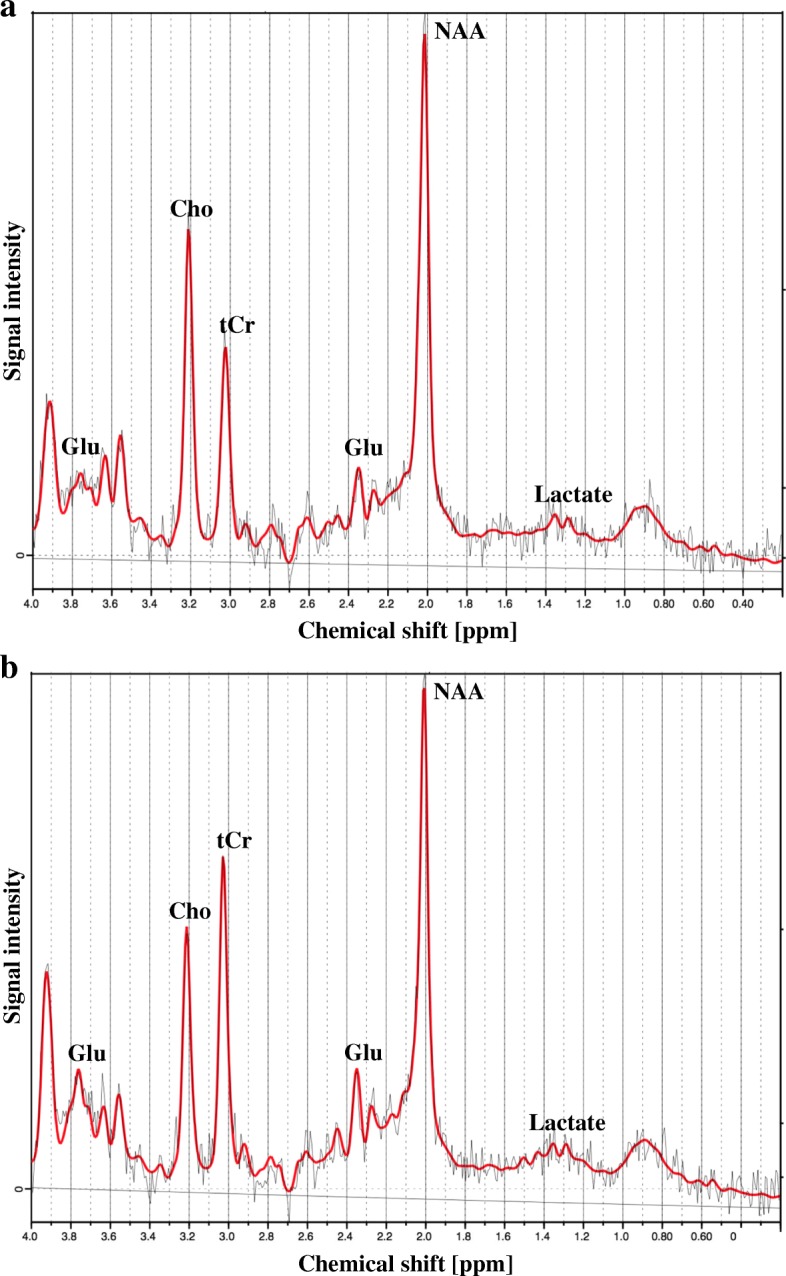
MR spectra from brainstem and thalamus. Examples of (**a**) brainstem and (**b**) thalamus spectra are obtained at baseline with the point-resolved spectroscopy (PRESS) pulse sequence at 3.0 T. The spectra are acquired from LCModel. The red line represents the fit, and the horizontal linear line represents the baseline as estimated by LCModel. Cho: Choline, Glu: Glutamate, tCr: Total creatine, NAA: *N*-Acetylaspartate

Post-processing and quantification of the spectral data were performed by LCModel (Version 6.3-1F, Toronto, Canada). Representative ^1^H-MRS spectra obtained from brainstem and thalamus are illustrated in Fig. 2. Spectra were evaluated in a blinded manner and abnormal spectra were excluded. The quality of the included spectra was estimated based on the signal-noise-ratio (SNR) and full-width of half-maximum (FWHM) of the spectra peaks as provided by LCModel. The means and standard deviations of the SNR and FWHM for the brainstem and thalamus spectra were calculated.

### Statistical analysis

The primary endpoint was glutamate, lactate, NAA and total creatine concentration changes in brainstem and thalamus from baseline to after sildenafil and CGRP administration, compared to the corresponding placebo changes. A linear mixed model was used for each metabolite with interaction between scans (baseline, scan 1 and scan 2) and drug days (sildenafil, CGRP and placebo) and with subjects and study day (5 levels) nested within subjects as random effects. The placebo day baseline scan was set as the reference parameter in the model.

The secondary endpoint was changes in the metabolite concentrations in participants who developed pharmacologically induced headache during the scan sessions after sildenafil and CGRP, compared to participants who did not. A linear mixed model was used for each metabolite on the sildenafil and CGRP day with interaction between scans and headache and the random effects: subjects and study day nested within subjects. The data did not allow for correlation analyses between metabolite concentration and headache characteristics. The headache frequencies after sildenafil and CGRP were compared to placebo using McNemar’s test.

For explorative vital parameter analyses, we included data from the following time points: 0, 20, 70, 120, and 170 min after infusion. Changes from baseline after sildenafil and CGRP were compared to placebo using a linear mixed model with interaction between drug days and the selected time points with subjects as random effects.

The variability structure of the glutamate measurements in the brainstem and thalamus was estimated in an explorative analysis based on baseline, scan 1, and scan 2 data acquired on the placebo day, and baseline data acquired on the sildenafil and CGRP day, using a linear mixed model with no fixed effects, and the random effects: subjects and study day (5 levels) nested within subjects.

All statistical analyses were performed using R (Version 3.4.2). *P* values were reported as two-tailed with a level of significance of 5%.

## Results

### Participants

**Fig. 3:**
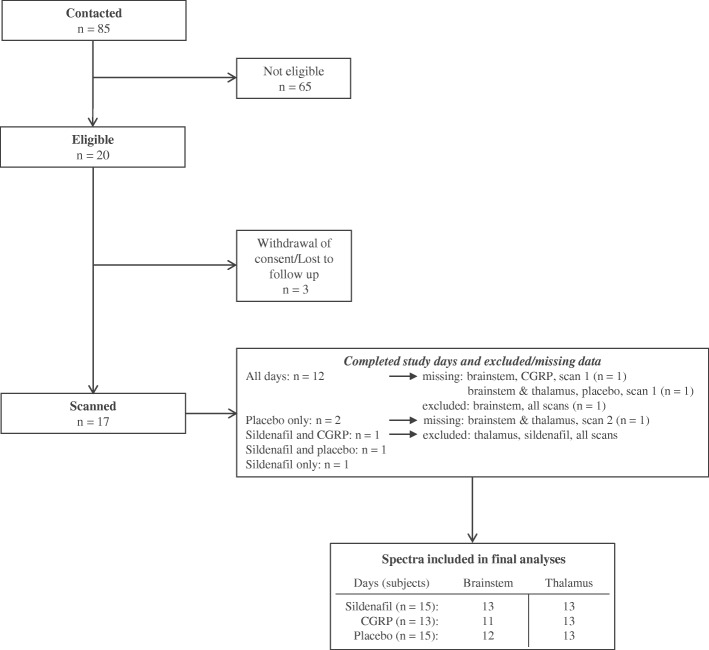
Flowchart of the inclusion process of participants and data for analyses. Seventeen participants were scanned, whereof 12 completed all three study days. The remaining participants completed 1–2 study days. One subject completed only the placebo day due to loss to follow up. One subject withdrew after scan 1 on the first study day (placebo) due to claustrophobia, thus scan 2 data are missing. One subject completed the sildenafil and CGRP day, but thalamus data were excluded from the sildenafil day as the subject fell asleep during the thalamus baseline scan. One subject did not complete the CGRP day due to loss to follow up. One subject only participated on the sildenafil day due to finalization of the study. Data were further missing due to technical issues: scan 1 data (brainstem and thalamus) after placebo from one subject, and scan 1 brainstem data after CGRP from another subject. Brainstem data were excluded from one subject for all three study days due to poor spectral quality. n: Number

**Fig. 4:**
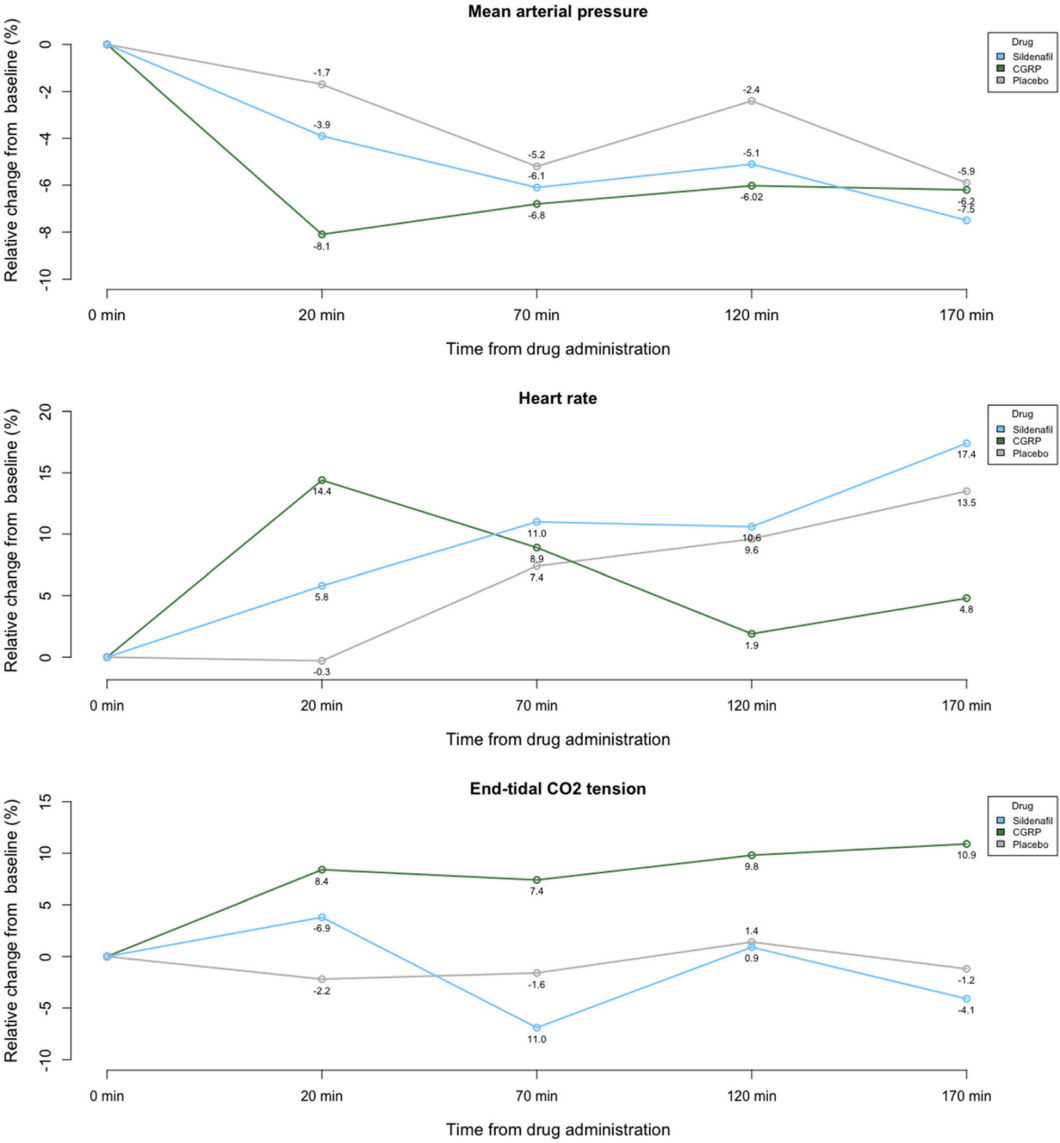
Mean arterial pressure, heart rate and end-tidal CO_2_ tension after sildenafil, CGRP and placebo. The mean arterial pressure, heart rate and end-tidal CO2 tension were normal on all three study days

Seventeen healthy volunteers participated in the study (10 women and 7 men) with mean age 22.9 (SD ± 3.4 and range 18–30 years) (Fig. 3). Vitals signs are presented in Fig. 4.

### Alterations in metabolite concentration

#### Brainstem

**Fig. 5:**
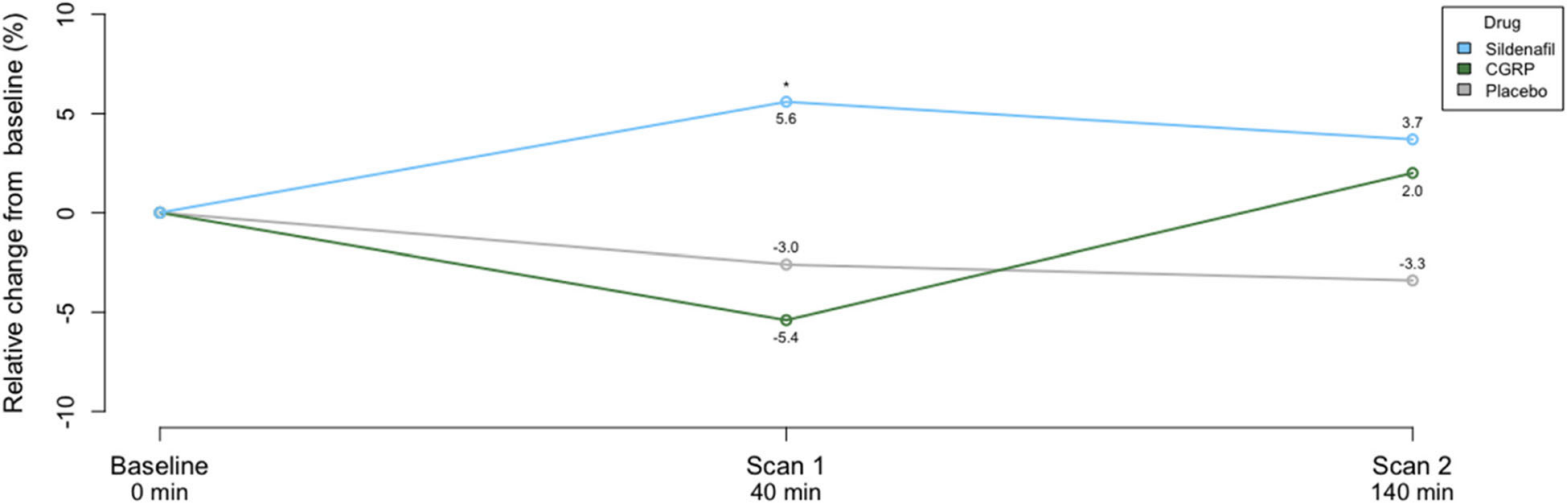
Mean glutamate changes after sildenafil, CGRP and placebo in brainstem . * *P* < 0.05, when compared to placebo

The glutamate concentration significantly increased from baseline to scan 1 after sildenafil compared to the corresponding change after placebo (*P* = 0.039) (Table 1, Fig. 5). The lactate concentration decreased from baseline to scan 1 (*P* = 0.017), but not to scan 2 (*P* = 0.156) after sildenafil, compared to corresponding changes after placebo. In the brainstem, we did not detect changes in the metabolite concentrations from baseline to scan 1 or scan 2 after CGRP, compared to placebo.

**Table 1:** Summary of metabolite concentrations in brainstem after sildenafil, CGRP and placebo

	Baseline		Scan 1				Scan 2			
	Mean mmol/L	SD	Mean mmol/L	SD	% change from baseline	*P*	Mean mmol/L	SD	% change from baseline	*P*
Glutamate										
Sildenafil	7.77	0.65	8.21	0.53	5.6	0.039*	8.06	0.77	3.7	0.101
CGRP	7.92	1.11	7.49	0.88	−5.4	0.639	8.08	0.73	−2.0	0.228
Placebo	7.96	0.50	7.72	0.61	−3.0	–	7.70	0.65	−3.3	–
Lactate										
Sildenafil	0.90	0.21	0.45	0.41	−50.0	0.017*	0.43	0.33	− 51.9	0.156
CGRP	0.71	0.51	0.49	0.44	−30.9	0.151	0.42	0.41	−40.6	0.494
Placebo	0.75	0.65	0.89	0.68	21.6	–	0.62	0.42	−15.6	–
NAA										
Sildenafil	7.73	0.79	7.93	0.79	2.8	0.236	7.95	0.69	3.0	0.370
CGRP	7.58	0.72	7.56	0.68	−0.2	0.821	8.06	0.82	6.3	0.127
Placebo	7.61	0.63	7.64	0.58	0.4	–	7.73	0.72	1.6	–
Total creatine										
Sildenafil	4.25	0.35	4.33	0.32	1.0	0.978	4.23	0.28	−0.3	0.735
CGRP	4.38	0.38	4.39	0.53	0.03	0.562	4.50	0.33	2.7	0.170
Placebo	4.24	0.33	4.33	0.47	2.0	–	4.21	0.32	−0.7	–

*P<0.05. *P* values reported for delta change from baseline to scan 1 and 2 after sildenafil and CGRP, compared to the corresponding change from baseline after placebo

*NAA N*-Acetylaspartate, *SD* standard deviation

#### Thalamus

We did not detect changes in the glutamate, lactate or NAA concentrations in the thalamus from baseline to scan 1 or scan 2 after sildenafil or CGRP, compared to placebo. The increase in the total creatine concentration from baseline to scan 2 after CGRP (3.3%, *P* = 0.028) was significant in comparison to placebo (*P* = 0.004).

### Headache vs. no headache

**Fig. 6:**
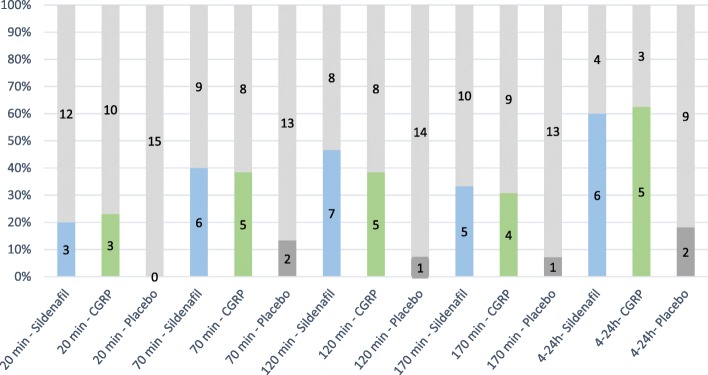
Proportion of healthy participants who developed headache after sildenafil, CGRP and placebo. Blue, green and dark grey bars indicate headache. Light grey bars indicate no headache. During the scan sessions (0–4 h), 8 of 13 participants (62%) developed headache after CGRP (*P* = 0.041, compared to placebo), 8 of 15 (53%) developed headache after sildenafil (*P* = 0.131, compared to placebo), and 2 of 13 (13%) developed headache after placebo.

The proportion of participants who developed headache during scan 1 and scan 2, and after the scan sessions and until 24 h from drug administration, is reported in Fig. 6. We found no interaction in the glutamate, lactate, NAA or total creatine concentrations in the brainstem or thalamus between participants who developed sildenafil- and/or CGRP-induced headaches as compared to participants who did not.

### Quality of spectra

The brainstem spectra had mean SNR of 17.56 (± 2.33), and mean FWHM of 0.05 ppm (± 0.01) / 6.39 Hz (± 1.28). In thalamus, the mean SNR was 15.30 (± 1.86) and the mean FWHM was 0.04 ppm (± 0.01) / 5.11 Hz (± 1.28). In addition, the Cramér–Rao lower bound was < 12% for glutamate measurements in the brainstem and thalamus, except for 12–13% in four brainstem spectra and one thalamus spectrum in different subjects.

### Glutamate variability in brainstem and thalamus

From the linear mixed model, we obtained separate brainstem glutamate concentration variations, where 6.9% was due to residual measurement error with additional 2.1% due to inter-subject variation, and 6.0% due to between day variations. The thalamic glutamate concentration variations were 6.8% due to residual measurement error with 2.7% inter-subject and 0% between day variations.

The mean time difference from day 1 to day 2 of the study days was 12.5 days (± 9.2) and 10.7 days (± 6.0) between day 2 and day 3. Participants were mainly scanned from afternoon time on all three scan days. The scans were initiated in the morning for three subjects, whereof one subject completed all three study days.

## Discussion

The major outcome of the present study was an increase in the glutamate concentration in the brainstem after administration of sildenafil when compared to placebo. We did not detect any changes in the glutamate concentration in the brainstem after CGRP infusion.

### Sildenafil-induced biochemical changes

Glutamate, as the major excitatory neurotransmitter in the brain, promotes neuronal depolarization [29]. Extracellular glutamate levels are directly correlated to levels of neuronal hyperexcitability and seizure intensity in animal models of epilepsy [30, 31]. Here, we evaluated the combined concentration of the glutamate and glutamine as these metabolites are not differentiable at 3.0 T ^1^H-MRS. In healthy volunteers, the majority of the combined concentration consists of glutamate (~ 80%) [32] and 13%–22% of the glutamate concentration in the healthy brain is present in the extracellular space [29]. Most likely, the glutamate concentrations measured by ^1^H-MRS largely reflect the extracellular glutamate levels. In support of this, a ^1^H-MRS study reported lower glutamate levels in amyotrophic lateral sclerosis patients treated with riluzole, a drug that increases glutamate uptake in central nervous system (CNS) neurons, compared to riluzole-naive amyotrophic lateral sclerosis patients and healthy controls [16]. The transient sildenafil-induced increase of glutamate in the brainstem in the present study thus likely reflects increased extracellular glutamate levels and possibly increased neuronal excitability. In support, sildenafil is able to cross the blood-brain barrier [23, 33] and some individuals report CNS side effects, such as dizziness and confusion [33–36]. Thus, sildenafil may be able to directly affect the neurons in deep brain structures such as the brainstem. In contrast, a functional MRI (fMRI) study of the visual cortex suggested that oral sildenafil intake did not change the neuronal activation threshold either at 1 or 2 h after administration [2]. The plasma t_max_ of oral 100 mg sildenafil is about 1 h with a close to 4 h half-life in the fasting state [36]. Here, we detected an increased glutamate level at scan 1 (40–70 min after sildenafil), around the time of t_max,_ but not at scan 2 (140–170 min after sildenafil). Possibly, plasma concentrations of sildenafil above a certain level are needed to alter the glutamate levels. Another possibility is that the transient changes may be attributed to adaptation of sildenafil’s effect at scan 2.

We detected no difference in the glutamate levels between groups of participants developing headache vs. no headache. It should be noted that the participants were healthy with no family history of migraine developing merely a mild to moderate non-migraine headache after the drug administration. Therefore, we speculate that a “healthy” trigeminonociceptive system would not be sufficiently activated to produce detectable changes in the glutamate level. This may also explain the lack of changes in the glutamate levels after CGRP as well as in the thalamus. Given the transient glutamate changes and lack of correlation to headache status, it is likely that the observed changes are related to the pharmacological effects of the drug rather than the headache per se.

The lactate concentration was decreased in the brainstem at scan 1 after sildenafil compared to the corresponding placebo change. This observation is very interesting since brain lactate levels under normal conditions increase during neuronal activation [37]. Therefore, we would expect the brainstem lactate levels to increase following sildenafil administration, along with the observed increase in glutamate. A possible explanation could be that the lactate decrease reflects a neuronal energy consumption via conversion to pyruvate [38]. The lactate concentration finding in the present study should be interpreted with caution due to the relatively large standard deviations. Also of note, the lactate concentration is very low in the healthy brain (below 1.0 mmol/L) [39]. This contributes to the risk of lactate signal loss in the spectrum due to chemical shift displacement or J-modulations deviations during the MRS measurements, which are known issues [39].

### CGRP-induced biochemical changes

We detected no alterations in the glutamate levels after CGRP infusion in either brainstem or thalamus in healthy participants. This suggests that CGRP does *not* modify the neuronal excitability in these key CNS structures involved in pain processing in healthy subjects. The blood-brain barrier is believed to have no permeability to CGRP [4, 40], and thus little or no direct effects on central brain regions, which our findings support.

In line with previous reports [3, 4], we found that participants developed more headache after CGRP, compared to placebo, demonstrating that CGRP is able to activate the trigeminal pain pathway. Given that systemic CGRP is unlikely to cross the blood-brain barrier, the present findings support the notion that CGRP acts on perivascular afferents [22] or the trigeminal ganglion [21, 41]. Interestingly, an fMRI study reported no change in the neuronal activation of the visual cortex of healthy volunteers after CGRP infusion [42]. Increased neuronal response to visual stimulation has previously been shown to be correlated with an increase in the glutamate levels [43, 44]. Another fMRI study involving application of heat pain to the forehead of healthy volunteers reported altered blood-oxygenation-level-dependent signal 40 min after administration of CGRP in pain associated brain regions, including the brainstem and thalamus, with no changes during placebo [45]. This observation suggests that CGRP may be capable of modulating the neuronal response indirectly (i.e. outside the CNS) given that the pain pathway is already activated [45].

### Reliability of glutamate measurements

With our ^1^H-MRS protocol, we obtained high quality spectra from the brainstem and thalamus with narrow line widths and relatively high SNR allowing for reliable quantification. A previous 3.0 T ^1^H-MRS study measured glutamate changes in the brainstem without reporting the SNR or spectral line widths, but visual inspection of the brainstem spectrum reveals more noise compared to the brainstem spectra obtained in the present study [16]. Other ^1^H-MRS brainstem studies reported relatively wider mean line widths of 8.1 Hz (± 0.9) [18], and 10.04 Hz (± 4.64) [17] at 3.0 T, and 7 Hz at 4.0 T [19] indicating spectra of lower quality. One of the studies reported the SNR as well, which was relatively high, 21(± 3), most likely due to the larger VOI used in the study [18]. None of the studies reported glutamate findings and the VOIs were larger than in the present study [17–19]. While large VOIs can improve the spectral quality, it also restricts the possibility of targeting a brain area with precision.

A previous 3.0 T ^1^H-MRS study of thalamus used a larger VOI with fewer acquisitions, and reported a mean line width similar to the present study findings, but did not report the SNR value for comparison [17]. One 1.5 T ^1^H-MRS thalamus study reported reduced mean line width of 3.2 Hz (± 0.5), however, the SNR of 3.9 (± 1.2) was much lower [46].

The brainstem ^1^H-MRS spectra reveal a different metabolite composition compared to the conventional spectra obtained from e.g. the thalamus and occipital lope, as the choline peak is higher than the total creatine peak (Fig. 2), which is commonly reported [16, 17, 19].

To our knowledge, our study is the first ^1^H-MRS study to provide information on the variability of glutamate levels in both brainstem and thalamus, based on repeated measurements, on the same day and on three separate days. In the present study, the overall variability of the glutamate measurements was low. For comparison, a previous 3.0 T ^1^H-MRS study reported a higher inter-subject glutamate variability of 15.4%–16.3% in the deep brain area of the amygdala, based on two scans obtained 1 week apart [47]. Another 3.0 T ^1^H-MRS study of repeated measurements on three consecutive days reported residual measurement error as the main contributor to the glutamate variability in a small hippocampus VOI [48]. Finally, one previous 7.0 T ^1^H-MRS study reported higher glutamate variability of 11.48% (± 8.87) within day (based on two scans), and 6.56% (± 4.69) between day, measured in the visual cortical area of healthy subjects [49]. However, the study did not report a separate inter-subject and residual measurement error variability [49].

The present study has several major strengths to account for the measurement error variation, as all participants were scanned at fixed time points on each scan day, accounting for possible changes due to the metabolic circadian rhythm [25, 26]. In addition, we maintained identical and stable study conditions for all participants on all three study days, including detailed dietary restrictions before and during the scan sessions. All participants were carefully instructed to avoid any head motion during the scans. However, we cannot exclude the possibility of motion affecting our findings during the scan sessions. We estimated that the high acquisition number for the ^1^H-MRS sequences was appropriate and feasible to obtain a sufficient signal noise ratio from the spectral VOIs. Additionally, as our primary aim was to investigate and compare relative changes from baseline within subjects, these issues were unlikely to affect our results.

## Conclusion

Here we present a protocol for pharmacological ^1^H-MRS at 3.0 T in the brainstem and thalamus, with good spectral quality, and overall low measurement variability. We demonstrated that sildenafil induces transiently increased glutamate levels in the brainstem, which suggest transiently increased excitability of the brainstem neurons. CGRP does not induce glutamate changes in the brainstem or thalamic neurons, suggesting that its headache-inducing effects are not mediated by biochemical changes in deep brain structures, but rather its effects on the peripheral trigeminal pain pathways.

## Abbreviations

^1^H: proton
CGRP: calcitonin gene-related peptide
CNS: central nervous system
fMRI: functional magnetic resonance imaging
FWHM: full-width of half-maximum
ICHD-3 beta: The diagnostic criteria of the beta version of the third International Classification of Headache Disorders
MRI: magnetic resonance imaging
MRS: magnetic resonance spectroscopy
NAA: *N*-Acetylaspartate
PRESS: point-resolved spectroscopy pulse sequence
SNR: signal-noise-ratio
VOI: volume-of-interest
